# Parasite Genotype Is a Major Predictor of Mortality from Visceral Leishmaniasis

**DOI:** 10.1128/mbio.02068-22

**Published:** 2022-10-12

**Authors:** Cooper Alastair Grace, Kátia Silene Sousa Carvalho, Mayara Ingrid Sousa Lima, Vladimir Costa Silva, João Luís Reis-Cunha, Matthew J. Brune, Sarah Forrester, Conceição de Maria Pedrozo e Silva de Azevedo, Dorcas Lamounier Costa, Doug Speed, Jeremy C. Mottram, Daniel C. Jeffares, Carlos H. N. Costa

**Affiliations:** a York Biomedical Research Institute, Department of Biology, University of York, York, United Kingdom; b Department of Community Medicine and Institute of Tropical Diseases Natan Portela, Federal University of Piauí, Teresina, Brazil; c Department of Biology, Postgraduate Programs in Health Sciences and Postgraduate Program in Health and Environment, Federal University of Maranhão, São Luís, Maranhão, Brazil; d Department of Medicine and Postgraduate Program in Health Sciences, Federal University of Maranhão, São Luís, Maranhão, Brazil; e Intelligence Center for Emerging and Neglected Diseases, Teresina, PI, Brazil; f Mother Child Department, Federal University of Piauí, Teresina, PI, Brazil; g Centre for Quantitative Genetics and Genomics, Aarhus University, Aarhus, Denmark; Albert Einstein College of Medicine

**Keywords:** visceral leishmaniasis, Brazil, *Leishmania infantum*, virulence factors, genetic diversity, quantitative genetics, mortality

## Abstract

Visceral leishmaniasis (VL) is a potentially fatal disease caused mainly by Leishmania infantum in South America and Leishmania donovani in Asia and Africa. Disease outcomes have been associated with patient genotype, nutrition, age, sex, comorbidities, and coinfections. In this study, we examine the effects of parasite genetic variation on VL disease severity in Brazil. We collected and sequenced the genomes of 109 L. infantum isolates from patients in northeastern Brazil and retrieved matching patient clinical data from medical records, including mortality, sex, HIV coinfection, and laboratory data (creatinine, hemoglobin, and leukocyte and platelet counts). We identified genetic differences between parasite isolates, including single nucleotide polymorphisms (SNPs), small insertions/deletions (indels), and variations in genic, intergenic, and chromosome copy numbers (copy number variants [CNVs]). To describe associations between the parasite genotypes and clinical outcomes, we applied quantitative genetics methods of heritability and genome-wide association studies (GWAS), treating clinical outcomes as traits that may be influenced by parasite genotype. Multiple aspects of the genetic analysis indicate that parasite genotype affects clinical outcomes. We estimate that parasite genotype explains 83% chance of mortality (narrow-sense heritability [*h*^2^] = 0.83 ± 0.17) and has a significant relationship with patient sex (*h*^2^ = 0.60 ± 0.27). Impacts of parasite genotype on other clinical traits are lower (*h*^2^ ≤ 0.34). GWAS analysis identified multiple parasite genetic loci that were significantly associated with clinical outcomes; 17 CNVs were significantly associated with mortality, two with creatinine, and one with bacterial coinfection, jaundice, and HIV coinfection, and two SNPs/indels and six CNVs were associated with age, jaundice, HIV and bacterial coinfections, creatinine, and/or bleeding sites. Parasite genotype is an important factor in VL disease severity in Brazil. Our analysis indicates that specific genetic differences between parasites act as virulence factors, enhancing risks of severe disease and mortality. More detailed understanding of these virulence factors could be exploited for novel therapies.

## INTRODUCTION

Leishmaniasis is a neglected tropical disease caused by protozoan parasites of the genus *Leishmania*. Visceral leishmaniasis (VL), the most severe form, is caused by Leishmania infantum in South America, the Mediterranean basin, the Middle East, and Central Asia and by Leishmania donovani in East Africa and the Indian subcontinent. VL is characterized by fever, weight loss, hepatosplenomegaly, anemia, pancytopenia, hypoalbuminemia, and hypergammaglobulinemia and is frequently fatal if untreated (1, 2). Severe disease presents as hepatic dysfunction with jaundice, edema, and dyspnea (3). Death is usually associated with hemorrhagic phenomena, which could be caused by bacterial coinfection (4, 5) as a consequence of cytokine-driven systemic inflammation (6). *Leishmania*/HIV results in greater risks of relapse and fatality (7, 8). Despite the range of clinical presentations, the parasite factors that determine differential virulence in humans are not well understood.

There are approximately 5,000 VL cases/year in Brazil, 90% of the cases registered in the Americas (9–12). There are large numbers of asymptomatic L. infantum infections, with various studies identifying 1 to 6% of the general population in regions of endemicity (7, 13, 14). VL case fatality rates in Brazil average 7%, the highest in the world, and show an increasing trend (12). Hence, understanding the factors that lead to severe disease and mortality is a priority for leishmaniasis research. Disease outcomes are known to be associated with patient age, sex, comorbidities (4), adverse effects of treatment, and coinfections such as HIV (15). Genome-wide association studies (GWAS) have shown to human genotypes influence VL disease severity in East Africa, Brazil, and the Indian subcontinent (16), including a strong influence from the HLA-DR-DQ region within the major histocompatibility complex (17). However, apart from studies of drug resistance (10, 18), the influence of natural parasite genetic variation on disease severity has not been investigated using genome-scale methods.

To investigate this, we collected 109 L. infantum isolates from the states of Piauí and Maranhão in Brazil, which have large VL burdens (19, 20). We used genome-scale quantitative genetic analyses to investigate the effects of parasite genotype on multiple clinical indicators of disease severity and mortality. Our analysis showed that parasite genotype influences multiple aspects of disease severity and has a strong relationship with patient mortality. We identified multiple parasite genetic loci that affect mortality. These virulence factors are key points for vaccine and drug discovery (21) that will be complementary to reverse vaccinology methods (22).

## RESULTS

### Sampling and clinical traits.

**FIG 1:**
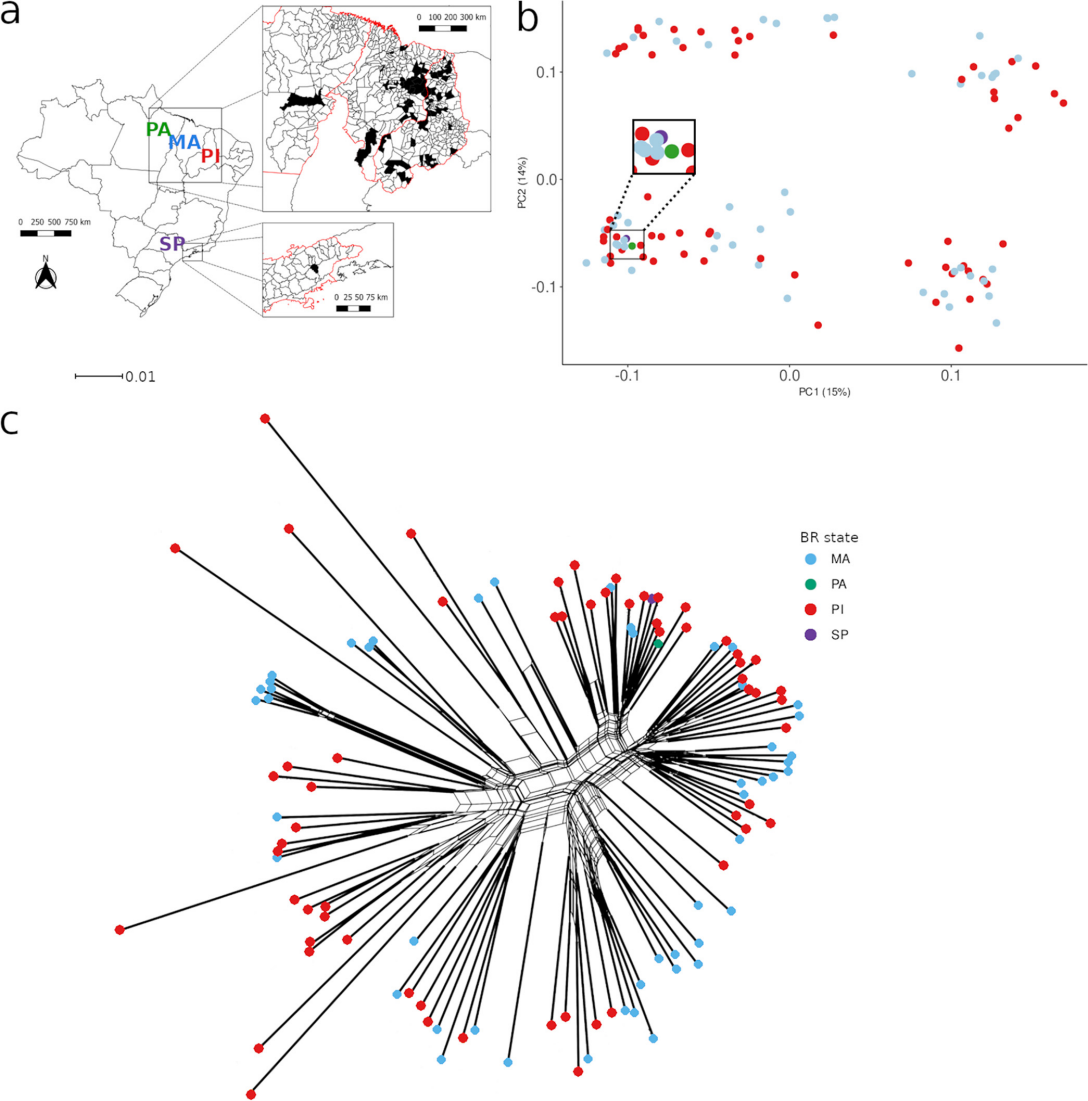
Sample origins and population structure. (a) Map showing residential locations of VL patients who provided the L. infantum strains. MA, Maranhão; PI, Piauí; PA, Pará; SP, São Paulo. Pará and São Paulo yielded a single isolate each. (b) Principal-component coordinates, derived from SNP variation, show that genetic relatedness is not clustered by state. Each filled circle indicates the principal-component-derived relatedness between isolates. Circles are colored by their state of origin. (c) Phylogenetic network analysis of all 109 strains, with colored circles indicating state of origin using the color scheme in panel b. Branches with thick lines have placement with 100% bootstrap support. The scale bar indicates the number of substitutions per site. The network file and version with isolate names are available at https://figshare.com/projects/Brazil-VL-GWAS/126331.

We collected L. infantum isolates for genomic sequencing and clinical data from 109 patients with VL in Brazil. The isolates were collected from patients in the states Piauí and Maranhão, where VL is highly endemic (Fig. 1a). Each parasite isolate was matched to the source patient with information on age; sex; the binary clinical factors mortality (recovered/died), bleeding, presence of edema, HIV status, jaundice, vomiting, dyspnea, and bacterial coinfection; laboratory clinical metrics (leukocyte and platelet counts and hemoglobin and creatinine levels); and estimated probabilities of mortality from clinical and clinical+laboratory models (Kala-Cal* and Kala-Cal** respectively) (5). All these factors varied among patients (see Fig. S13 at https://figshare.com/projects/Brazil-VL-GWAS/126331; see Tables S2 and S3 in the supplemental material), and mortality was well predicted by the Kala-Cal* model (see Fig. S14 at the address above). In this cohort, no trait, except age, was associated with HIV status (see Fig. S15 at the address above). The only other between-trait correlations were that leukocyte and creatinine counts declined with patient age (see Fig. S16 at the address above).

10.1128/mbio.02068-22.2 TABLE S2 Phenotypic trait summary. Download Table S2, XLSX file, 0.05 MB. Copyright © 2022 Grace et al. 2022 Grace et al. https://creativecommons.org/licenses/by/4.0/ This content is distributed under the terms of the Creative Commons Attribution 4.0 International license.

10.1128/mbio.02068-22.3 TABLE S3 Patient phenotypic data. Download Table S3, XLSX file, 0.01 MB. Copyright © 2022 Grace et al. 2022 Grace et al. https://creativecommons.org/licenses/by/4.0/ This content is distributed under the terms of the Creative Commons Attribution 4.0 International license.

### Genomic variation.

To characterize genomic variation in these 109 L. infantum isolates, we identified single nucleotide polymorphism (SNP) and insertion/deletion (indel) variants, gene copy number variations (GCNVs), intergenic copy number variations (IGCNVs), and chromosome copy number variations (CCNVs) (Table 1). In accordance with other analyses (19, 23, 24), L. infantum populations contain 10-fold less genetic diversity than L. donovani populations in East Africa (nucleotide diversity [π] in Brazil = 4.3 × 10^−5^; Africa π = 42 × 10^−5^) and half as much diversity as the Indian subcontinent (India π = 8 × 10^−5^). Analysis of between-strain relatedness with principal-component analysis (PCA) and phylogenetics showed very little population structuring, with no clear distinction between isolates based on state of origin (Fig. 1b and c). The majority of chromosomes were disomic (see Fig. S2 and S3 at https://figshare.com/projects/Brazil-VL-GWAS/126331), whereas a high number of chromosomal duplications and GCNVs were observed in several isolates, as expected for *Leishmania* and other eukaryotic genomes (10, 25).

**TABLE 1:** L. infantum genomic variability of 109 isolates

Genomic variation	Count
SNVs^*a*^	14,203
SNPs	4,468
Indels	9,735
Variant sites with MAF >5%	3,526
Biallelic variants	3,004
Genes	8,748
Genes with CV of >1% and coverage at >20% of genome coverage	8,218
Intergenic regions	8777
Intergenic regions with CV of >1% and coverage at >20% of genome coverage	8,219

a SNVs, small nucleotide variants; MAF, minor allele frequency; CV, coefficient of variation.

### Effects of genetic variation in Leishmania infantum on clinical factors.

To quantify the effect of parasite genetic variation, we estimated the narrow-sense (additive) heritability, using clinical factors as traits (Table S2) and parasite genetic variants as genotypes (see Materials and Methods). This analysis estimated the proportion of variation in patient clinical factors that is attributed to parasite genetic variation. Possible kinship between parasites was inferred by LDAK using genetic variants (see Materials and Methods). We utilized four categories of genetic variants in this analysis: (i) SNP/indel variants, (ii) CCNV, (iii) GCNV, and (iv) IGCNV. Heritability estimation was performed using each genotype category independently, followed by a composite model that estimates the combined contribution of all variants. As we sought to examine the effects of parasite variation, host genotypes were not considered in this analysis.

**FIG 2:**
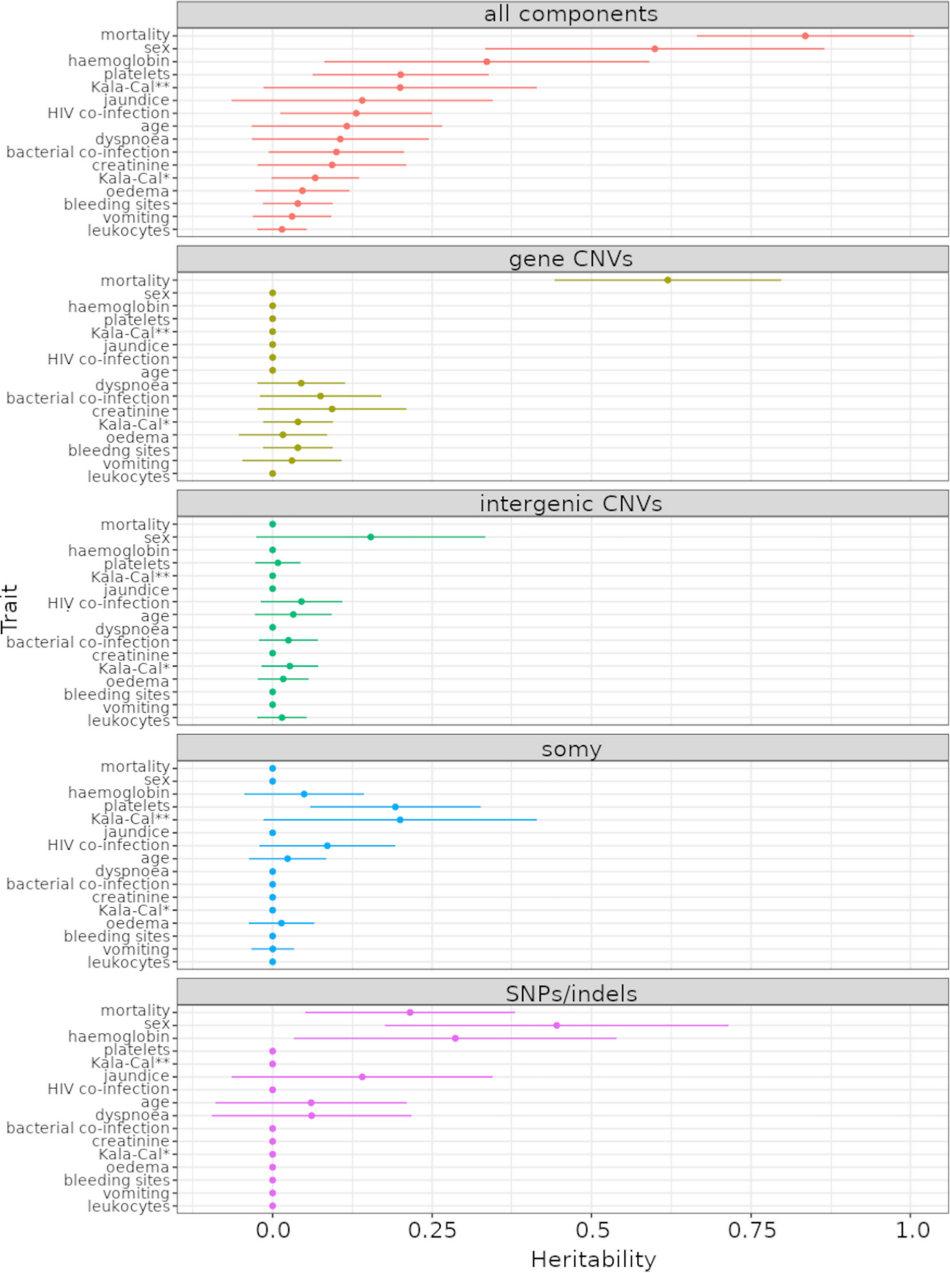
Heritability estimates from the composite model. Heritability estimates used normalized trait values. Horizontal lines represent standard deviations for each heritability estimate. Here, most analyses used 109 samples. (Top) Estimate of total heritability from all types of genetic variants combined. Panels below this show the heritability contributed by the various types of genetic variants. Due to linkage between different categories of variants (SNPs, gene copy variants, etc.), the total heritability explained is not expected to be the sum of all variant types.

Our results show that parasite genotype has significant effects on multiple clinical factors (Fig. 2). Due to the small sample size (*n *= 109), all estimates have relatively large standard deviations (Table S4). Mortality was strongly affected by parasite genetic variation, with a total heritability of 0.83 (standard deviation, 0.17; 95% confidence interval, 0.66 to 1.00), impacted by SNPs/indels and GCNVs. Sex was also strongly associated with SNP/indel variation (0.60 ± 0.27; 95% confidence interval, 0.33 to 0.87), indicating that parasite genotype influences the susceptibility of males and females to VL differentially. Strain-specific differences that affect male and female disease severity may be the cause of the higher occurrence of infections in males (15). The majority of the other traits were affected primarily by SNP/indel variations, rather than CNVs. Approximately a third of clinical traits (*n *= 5) are affected by independent heritability components, while the majority of traits (*n *= 11) are complex, with multiple types of genotypic variation contributing to host phenotype. This analysis shows that even within Brazil, where L. infantum genetic diversity is relatively low, differences between parasites influence disease progression.

10.1128/mbio.02068-22.4 TABLE S4 Heritability values. Download Table S4, XLSX file, 0.01 MB. Copyright © 2022 Grace et al. 2022 Grace et al. https://creativecommons.org/licenses/by/4.0/ This content is distributed under the terms of the Creative Commons Attribution 4.0 International license.

The effect of parasite genotype on mortality could be due to one lineage of particularly virulent parasites. To assess this possibility, we visualized this clinical outcome on a phylogenetic tree of isolates (see Fig. S4 at https://figshare.com/projects/Brazil-VL-GWAS/126331). Isolates from patients who died are dispersed throughout the phylogeny, indicating that no specific lineage is responsible for mortality. This is expected, if multiple loci affecting mortality were segregating within the population and these loci are unlinked by recombination between strains (see Fig. S1 at https://figshare.com/projects/Brazil-VL-GWAS/126331). In this scenario, no single phylogeny will cluster these unlinked variants.

To identify the genetic loci that affect clinical outcomes, we conducted GWAS, using all four categories of genetic variants as predictors of clinical traits. In all analyses apart from chromosome copies (CCNVs), association *P* values were inflated above expected values, indicating that multiple genetic loci affect clinical outcomes (QQ plots, Fig. S5 and S12 at https://figshare.com/projects/Brazil-VL-GWAS/126331). We observed no association between CCNV and any of the evaluated traits, which is in accordance with chromosome copy number variation (somy) being associated only with short-term environmental adaptation (26) that may vary after culturing (27). Given the small sample size available (*n *= 109), we expect to discover only cases where genotypes have strong effects on clinical outcomes. Nevertheless, we found multiple parasite genetic variants that were associated with clinical outcomes above our permutation-derived thresholds (Table 2; Tables S5, S6, and S8).

**TABLE 2:** Genomic variations associated with clinical traits

GWAS component	No. of significant associations	Trait(s)
SNPs/indels	2	Age; jaundice
GCNVs	11	Mortality; creatinine; jaundice
IGCNVs	12	Bacterial coinfection; creatinine; mortality; HIV coinfection
CCNVs	0	

10.1128/mbio.02068-22.5 TABLE S5 Significant SNP/indel variant associations discovered with GWAS and annotations. Download Table S5, XLSX file, 0.1 MB. Copyright © 2022 Grace et al. 2022 Grace et al. https://creativecommons.org/licenses/by/4.0/ This content is distributed under the terms of the Creative Commons Attribution 4.0 International license.

10.1128/mbio.02068-22.6 TABLE S6 Significant gene CNV associations discovered with GWAS. Download Table S6, XLSX file, 0.01 MB. Copyright © 2022 Grace et al. 2022 Grace et al. https://creativecommons.org/licenses/by/4.0/ This content is distributed under the terms of the Creative Commons Attribution 4.0 International license.

10.1128/mbio.02068-22.8 TABLE S8 Significant intergenic CNV associations discovered with GWAS. Download Table S8, XLSX file, 0.01 MB. Copyright © 2022 Grace et al. 2022 Grace et al. https://creativecommons.org/licenses/by/4.0/ This content is distributed under the terms of the Creative Commons Attribution 4.0 International license.

**FIG 3:**
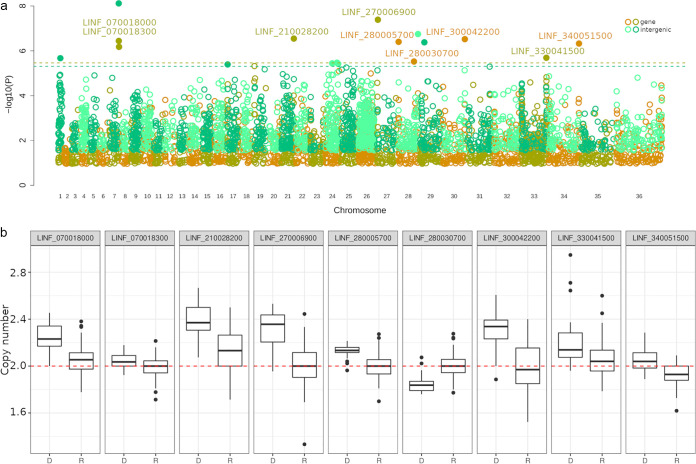
Association between genetic variation and mortality. (a) Manhattan plot displaying significant associations between GCNVs (yellow/orange), IGCNVs (dark/light green), and the mortality phenotype. Horizontal dashed lines indicate significance thresholds, calculated with 1,000 permutations (see Materials and Methods). Significant variants are highlighted by filled circles. Significant GCNVs are also named. −Log_10_ *P* values were filtered to remove the 50% least significant values to aid figure clarity. (b) The copy numbers of significant GCNVs are displayed as box plots, indicating copies from isolates where the patient either died (D) or recovered (R). The dashed line indicates expected disomy. Mann-Whitney tests confirmed that copy numbers for these genes are significantly different in parasites causing patient mortality: LINF_070018000, *P = *1.26 × 10^−7^; LINF_070018300, *P = *2.99 × 10^−3^; LINF_210028200, *P = *1.60 × 10^−8^; LINF_270006900, *P = *1.17 × 10^−8^; LINF_280005700, *P = *1.89 × 10^−8^; LINF_280030700, *P = *5.76 × 10^−8^; LINF_300042200, *P = *4.59 × 10^−8^; LINF_330041500, *P = *1.45 × 10^−3^; LINF_340051500, *P = *1.58 × 10^−7^.

Two small variants were significantly associated with clinical factors. An indel variant was significantly associated with the presence of jaundice, and a SNP variant was associated with patient age (*P = *5.2 × 10^−7^ and *P = *2.6 × 10^−6^) (Table S5; also, see Fig. S5 at https://figshare.com/projects/Brazil-VL-GWAS/126331). Both of these variants were located within intergenic regions (see supplemental text B at the address above). Gene copy variants were significantly associated with three traits: mortality (Fig. 3), creatinine levels, and jaundice (see Fig. S7 at the address above). Variations in intergenic DNA copies were associated with four clinical factors: bacterial coinfection, creatinine, mortality and HIV coinfection (see Fig. S8, S9, and S21 to S23 in supplemental text C at the address above). The association scores, chromosomal positions, and annotation of the 11 significant genes and 12 intergenic regions are displayed in Tables S6 and S8, respectively. As expected, given the evidence for recombination in these isolates, we found that some strains contain multiple virulence-associated genotypes and others contain only a few (Table S7), which may result in a range of virulence levels.

10.1128/mbio.02068-22.7 TABLE S7 Isolates by virulence, according to CNVs. Download Table S7, XLSX file, 0.1 MB. Copyright © 2022 Grace et al. 2022 Grace et al. https://creativecommons.org/licenses/by/4.0/ This content is distributed under the terms of the Creative Commons Attribution 4.0 International license.

The progression of disease is likely to be influenced by interactions between patient factors, comorbidities, and parasite genotype. For example, it is possible that particular parasite genotypes are more likely to infect HIV-positive patients that are immunosuppressed. To examine this possibility, we conducted analysis of gene and intergenic copy variants that affect mortality, using all other clinical traits as covariates. This analysis frequently produced lower (more significant) association *P* values (Tables S9 and S10). The most significant association using covariates was between the gene LINF_340051500 (phosphatidylinositol 3-kinase; *tor2*) copy variant and mortality (*P = *6.24 × 10^−9^), using Kala-Cal** risk prediction as a covariate. Since the Kala-Cal** mortality prediction uses many clinical characteristics, it likely captures many factors of the relationship between clinical factors, parasite, and mortality. The gene LINF_270006900 (hypothetical protein) was still significantly associated with mortality when each other trait was individually used as a covariate and when all the other traits excluding Kala-Cal* and Kala-Cal** were simultaneously used as covariates (Table S9). While these results do not define the precise relationships between parasite, host, and factors, they show that genetic association studies have considerable promise.

10.1128/mbio.02068-22.9 TABLE S9 Covariate analysis of gene CNVs. Download Table S9, XLSX file, 0.01 MB. Copyright © 2022 Grace et al. 2022 Grace et al. https://creativecommons.org/licenses/by/4.0/ This content is distributed under the terms of the Creative Commons Attribution 4.0 International license.

10.1128/mbio.02068-22.10 TABLE S10 Covariate analysis of intergenic CNVs. Download Table S10, XLSX file, 0.01 MB. Copyright © 2022 Grace et al. 2022 Grace et al. https://creativecommons.org/licenses/by/4.0/ This content is distributed under the terms of the Creative Commons Attribution 4.0 International license.

## DISCUSSION

We used genome data from 109 L. infantum isolates from northeastern Brazil coupled with detailed patient clinical data to study the effects of parasite variation on VL disease. We found that the chance of mortality and patient sex were strongly associated with parasite genotype (heritability, 0.83 ± 0.17 and 0.60 ± 0.27, respectively). Although our sample size is very small, we found multiple associations that are supported by permutation-derived thresholds for several clinical traits that are not correlated with one another. Both these significant GWAS associations (Fig. 3) and the inflation of *P* values in the SNP QQ plots indicate that multiple genetic factors contribute to different aspects of clinical outcomes. While this sample size is very small, similarly small sample sizes have identified significant association with microbial GWAS (28, 29).

We expect that disease outcomes and clinical manifestations are a combination of the interaction between the parasite, patient, environmental factors, and treatment. While no study has investigated the effects of human genetic variation on disease severity or mortality, several studies have evaluated the effects of human genetic variation on both visceral and cutaneous leishmaniasis susceptibility using GWAS (17).

The VL analysis identified a single locus with high significance; the human leukocyte antigen (HLA) haplotypes in the HLA-DR-DQ region (*P = *2.76 × 10^−17^) were the only genetic determinant of susceptibility to VL (17) that was consistent between populations in Brazil and India. Much weaker association hits (top hits at a *P* value of <5 × 10^−5^) were observed for susceptibility to cutaneous leishmaniasis (CL). Our analysis of parasite genetic variants is entirely consistent with these results, if we consider that host-parasite interactions determine clinical severity. The most striking result of our analysis is the magnitude of the contribution from the parasite genotype that we detected, without considering the human genotype. We consider this result reasonable from two perspectives. First, the effects of human genotypes on disease severity have not been examined directly (only disease susceptibility), and second, it is the parasite that causes disease, while host genotypes merely react to, and modify, disease severity.

Parasite-derived drug resistance and drug pharmacokinetics are also important factors (30). Genome-scale approaches have identified loci in L. donovani that are associated with antimonial resistance, which affect clinical outcomes (31, 32). Similarly, the low efficacy of miltefosine in Brazil, compared to initial efficacies in the Indian subcontinent, has been associated with the deletion of the miltefosine sensitivity locus (MSL), which that has been detected only in L. infantum isolates from Brazil (10). In our analysis, we did not observe any effects of MSL gene deletions on mortality or any other trait (GWAS *P* values for mortality for all MSL genes > 1 × 10^−2^), consistent with MSL being related to miltefosine resistance rather than mortality. The lack of association between the MSL locus and mortality is expected for our data set, as none of the evaluated patients were treated with miltefosine.

We consider it unlikely that the associations of mortality and sex with parasite genotype described here are due to drug resistance. This cohort is not ideal for such an analysis, since patients were treated with a variety of drugs, depending on disease severity (meglumine antimoniate, amphotericin B, liposomal amphotericin B, or pentamidine) (Table S3). Deaths from VL usually happen in the first week of admission. Drug treatments, even when resistance is present, are effective at this point, and resistance is noted only weeks after the beginning of the therapy. At present, no clear relationship between treatment failure and susceptibility to these drugs has been demonstrated in Brazil. Preliminary *in vitro* susceptibility analysis with some of the L. infantum isolates examined here confirms the lack of association between patient mortality and parasite drug susceptibility (M. Lima, unpublished data). However, host sex is implicated in VL incidence (33, 34) and increased parasitic load (35) but not in host mortality (4, 5). Therefore, the links of L. infantum genome to mortality and sex seems to follow distinct pathogenic pathways.

Our GWAS analysis indicated that a variety of different genetic variants were associated with clinical outcomes (Table 2). We discuss the potential implications of each observed SNP/indel and GCNV in supplemental texts B and C (https://figshare.com/projects/Brazil-VL-GWAS/126331), respectively. While we estimate that the parasite genotype has a strong influence on the chance of mortality (*h*^2^ = 0.83 ± 0.17; 95% confidence interval, 0.66 to 1.00), and some proportion of this effect appears to be derived from SNPs (Fig. 2), we did not observe any SNPs that were significantly associated with mortality (Fig. 3). This is possibly due to mortality being influenced by multiple alleles, each with small effects. While the significance values in our GWAS analysis appear fairly modest compared to GWAS analysis of human genetic variants that affect VL susceptibility (17), several considerations need to be taken into account. First, we have a smaller sample size (*n *= 109), so we cannot expect such strongly significant associations from a GWAS. Our results remain significant after permutation, because the *Leishmania* genome is smaller than human/dog genomes, which reduces the burden of multiple test correction. Nevertheless, some of our associations are very strong, particularly when covariate analysis is carried out. The gene LINF_340051500 was supported by a robust *P* value of 6.4 × 10^−9^ when Kala-Cal** was used as a covariate. Similarly, the genes LINF_270006900 and LINF_330042400 were supported when each trait besides Kala-Cal* and Kala-Cal** was simultaneously used as a covariate (*P* values of 5.9 × 10^−7^ and 1.19 × 10^−6^, respectively) (Table S9), and LINF_270006900 was also supported in all cases when each other trait was individually used as a covariate (*P* values ranging from 8.08 × 10^−7^ to 1.25 × 10^−8^) (Table S9).

Many of the variants associated with clinical outcomes defy mechanistic explanation. This is unsurprising given the poor state of basic knowledge about *Leishmania*-host interactions. Intergenic copy variants are particularly challenging to explain mechanistically, as the function(s) of intergenic regions in *Leishmania* genomes is not well understood. However, intergenic regions in the human genome and yeast genomes are frequently associated with phenotypes, consistent with known functional elements within intergenic regions of these species (29, 36).

We found no associations between chromosome number (CCNVs) and phenotypic traits. This is in accordance with previous work suggesting that karyotypic levels change rapidly between insect and mammalian hosts and also when parasites are cultured, while GCNVs are drivers of long-term adaptation in the field (26). As the DNA from the parasites was obtained after only two passages, and significant changes in parasite virulence due to culturing are observed only after 20 to 30 passages (37), we believe that SNPs and GCNV alterations due to culturing would be minimal. Furthermore, even if small (SNP/indel) variations are altered within cultured parasites, such variations were previously associated with a reduction in virulence, which is restored with passages in mice (37, 38). Hence, genetic changes with parasite culture would be expected to reduce the strength of our GWAS/heritability associations between parasite genotype and clinical outcomes, rather than create artifactual ones. The strong relationships between parasite genotype and clinical factors observed in the present work indicate that the genome data obtained from cultured parasites relate meaningfully to outcomes.

We envisage several implications of our results. Most importantly, the strong influence of parasite genetic variation on mortality indicates that L. infantum isolates vary in their virulence, so the risk of mortality depends at least in part on the isolate that causes the infection. Variation in parasite virulence within Brazilian L. infantum isolates may explain the range of clinical VL symptoms, from symptomatic infections (39), to rare occurrences of highly virulent infections that result in a high fatality rate for VL in Brazil (40). While it may be possible to use parasite genotypes as early predictors of severe infections, this is unlikely to be economically viable or practically feasible at present, given the complexity of the genotypes associated with mortality and the requirement for parasite culture. It is more practical to use clinical data for risk assessments, which are well-powered and feasible in resource-poor settings (5) (see Fig. S14 at https://figshare.com/projects/Brazil-VL-GWAS/126331). However, studies of this type will lead to an enhanced understanding of the molecular mechanisms that lead to severe VL disease. The pathway to this understanding will be a combination of assessing genetic markers, the parasite genes that are implicated, and the host genes enrolled in the immune responses that the different alleles elicit. As many *Leishmania* genes lack detailed gene functional annotations, laboratory analysis will be required to obtain mechanistic insights.

## MATERIALS AND METHODS

### Study design and sample collection.

The objective of this study was to search for relationships between parasite genotypes and patient clinical factors and outcomes, including mortality. To gather parasite genotypes, we collected L. infantum parasite samples from 109 diagnostic aspirates of 109 patients in the states of Piauí and Maranhão (Fig. 1), cultured them, and sequenced their genomes. DNA extraction from promastigote parasites was performed after two culture passages, to minimize the impact of culture adaptation. Each parasite genome is matched to clinical patient data. The corresponding patient and clinical data were extracted from medical records and routine patient follow-up. This included patient factors (age and sex), clinical characteristics (mortality, bleeding, edema, HIV coinfection, jaundice, vomiting, dyspnea, and bacterial coinfection), and laboratory clinical metrics (leukocyte and platelet counts and creatinine and hemoglobin levels). We also estimated probabilities of mortality from clinical or clinical + laboratory models (Kala-Cal* and Kala-Cal**) (Tables S1 and S2) (5). Our data analysis employed quantitative genetics methods of narrow-sense (additive) heritability and genome-wide association studies (GWAS) to associate specific parasite genetic variants (SNPs, indels, and gene and chromosomal CNVs) with patient factors and clinical characteristics.

10.1128/mbio.02068-22.1 TABLE S1 Isolates used in the study. Download Table S1, XLSX file, 0.02 MB. Copyright © 2022 Grace et al. 2022 Grace et al. https://creativecommons.org/licenses/by/4.0/ This content is distributed under the terms of the Creative Commons Attribution 4.0 International license.

### Ethics.

Piauí samples and patient data were obtained as a part of a broad study from the Federal University of Piauí and Federal University of Maranhão, respectively. Patient recruitment was performed at the Institute of Tropical Medicine Natan Portella, approved by the Research Ethics Committee of the Federal University of Piauí (approval ID 0116/2005), and in the Reference Hospital for Infectious Diseases of the Maranhão, approved by the Research Ethics Committee of the University Hospital of the Federal University of Maranhao (approval ID 2.793.599) and the Research Ethics Committee of the Federal University of Maranhao (approval ID 3.921.086). Both projects were approved by the Department of Biology Ethics Committee at the University of York (approval IDs DJ201803 and DJ202009). All methods were performed according to approved guidelines and regulations. All participants or their legal guardians signed a written informed consent form. Clinical, epidemiological, and laboratory data from patients were obtained during hospital admission or medical follow-up or from medical records. All patients were treated with one of the following protocols, based on disease severity: (i) pentavalent antimony (20 mg/kg/day; maximum, 800 mg/day) for 21 days, (ii) amphotericin B deoxycholate (1 mg/kg/day for 14 to 20 days), or (iii) liposomal amphotericin B (AmBisome; 3 mg/kg/day for 7 days for immunocompetent and 14 days for immunocompromised patients). Parasites were isolated from bone marrow aspirates before the start of patient treatment, and culturing was performed at the Laboratory of Leishmaniasis at the Universidade Federal do Piauí or at the Laboratory of Genetics and Molecular Biology at the Universidade Federal do Maranhão.

### Sequencing and bioinformatic analyses.

Genome sequencing was performed on Illumina HiSeq 2500, generating paired-end 150-nucleotide (nt) reads. All but eight of the libraries had genome coverage of ≥30× (median, 49×). (Table S1). Sequencing of 73 isolates was described previously (23). Sequencing reads were mapped onto the L. infantum JPCM5 v.46 (https://tritrypdb.org) reference genome using bwa v.0.7.17 (41). Read duplicates were removed with SAMtools v.1.9 (42). For each strain, SNPs and indels were called using Genome Analysis Toolkit (GATK) program HaplotypeCaller v.4.1.0.0 (43) and the “discovery” genotyping mode of Freebayes v.1.3.2 (https://github.com/ekg/freebayes), accepting only calls discovered by both methods. We retained only biallelic variants, with read depth within 0.3 to 1.7× the chromosome coverage, excluding any variants called on repetitive regions (see the supplemental methods at https://figshare.com/projects/Brazil-VL-GWAS/126331). All genomes were initially compared using phylogenetic approaches and PCA (see the supplemental methods). The gene, intergenic, and chromosome copy number variation of the 109 isolates was also assessed. This was performed by comparing variations in read depth coverage (RDC) between isolates (see the supplemental methods).

### Genetic analysis; estimating heritability and GWAS.

Heritability analysis is traditionally performed by analyzing individuals for whom we have pedigree information. The basic principle is that if a trait is heritable, then pairs of individuals who are more closely related (i.e., more genetically similar) will tend to be more phenotypically similar than pairs who are distantly related (i.e., less genetically similar). This enables us to estimate heritability based on the phenotypic correlations between twins, full siblings, parent-offspring pairs, etc. The advent of SNP genotyping means that it is no longer necessary to know the pedigree of the individuals, as family relationships can instead be inferred from SNP values. Further, it becomes possible to include information from unrelated pairs of individuals (in which case, the analysis leverages the fact that even across unrelated individuals, some pairs will by chance be more genetically similar than others).

SNPs/indels and gene, intergenic region, and chromosome CNVs were used as genotypes to estimate the total effect of parasite genetic variation on the patient clinical traits (narrow-sense heritability), using LDAK v.5.1 (44). In this analysis, clinical data were coded as either binary or quantitative traits (Table S2). PLINK v.1.9 (45) was used to create the binary files. Gene, intergenic, and chromosome CNVs were generated from RDC values normalized by genome coverage for each gene, intergenic region, or chromosome and coded as quantitative genotypes. Gene CNVs varied from 0 to 3.8 copies per haploid genome copy (mean, 1.017). The contribution of genotype class to each clinical trait was estimated using LDAK restricted maximum likelihood (REML) (see the supplemental methods at https://figshare.com/projects/Brazil-VL-GWAS/126331).

We used GWAS to identify specific genomic variants that were statistically associated with each clinical trait. The LDAK function –linear was used to conduct mixed-model GWAS, using the kinship matrix derived from SNP/indel variants to control for unequal strain relatedness. Significance thresholds were determined via trait permutation. For each trait, values were permuted 1,000 times, and parasite genotype-human trait associations were estimated. The lowest *P* value was determined from each replicate permutation analysis, and the 5th percentile was used as the significance threshold for associations (i.e., 5% family-wise false discovery rate) for each trait. *P*-value thresholds varied from 5.2 to 7.3 −log_10_. GWAS for SNPs/indels used only biallelic sites with call quality of ≥2,000 and population-wide minor allele frequency (MAF) of >5% across all 109 isolates (i.e., an allele must be present in at least 11 of the 218 haploid chromosomes to be used; *n *= 3,526). GWAS for CNVs used only genes with a mean genome coverage of >20% and a coverage coefficient of variation (CV) (SD/mean) of >1% (*n *= 8,219 genes; see the supplemental methods at https://figshare.com/projects/Brazil-VL-GWAS/126331).

### Data availability.

Genome sequencing data produced here are available from NCBI’s sequencing read archive under the BioProject ID PRJNA781413. All other sequencing data used are listed in Table S1. The supplemental material, including scripts used to run associations and permutations as well as the script to generate the Manhattan, QQ, correlation, and box plots and the alignments and resulting phylogenetic tree files, is available at https://figshare.com/projects/Brazil-VL-GWAS/126331.
